# FAM3B (PANDER) functions as a co‐activator of FOXO1 to promote gluconeogenesis in hepatocytes

**DOI:** 10.1111/jcmm.14073

**Published:** 2018-11-28

**Authors:** Yujing Chi, Yuhong Meng, Junpei Wang, Weili Yang, Zhe Wu, Mei Li, Di Wang, Fangfang Gao, Bin Geng, Lu Tie, Weiping Zhang, Jichun Yang

**Affiliations:** ^1^ Department of Central Laboratory & Institute of Clinical Molecular Biology Peking University People’s Hospital Beijing China; ^2^ Department of Physiology and Pathophysiology, School of Basic Medical Sciences Key Laboratory of Cardiovascular Science of the Ministry of Education, Center for Non‐coding RNA Medicine, Peking University Health Science Center Beijing China; ^3^ Department of Gastroenterology Peking University People's Hospital Beijing China; ^4^ State Key Laboratory of Cardiovascular Disease Hypertension Center, Fuwai Hospital, CAMS and PUMC, National Center for Cardiovascular Diseases Beijing China; ^5^ Department of Pharmacology, School of Basic Medical Sciences Peking University Beijing China; ^6^ Department of Pathophysiology Second Military Medical University Shanghai China

**Keywords:** co‐activation, FOXO1, gluconeogenesis, PANDER

## Abstract

FAM3B, also known as PANcreatic DERived factor (PANDER), promotes gluconeogenesis and lipogenesis in hepatocytes. However, the underlying mechanism(s) still remains largely unclear. This study determined the mechanism of PANDER‐induced FOXO1 activation in hepatocytes. In mouse livers and cultured hepatocytes, PANDER protein is located in both the cytoplasm and nucleus. Nuclear PANDER distribution was increased in the livers of obese mice. In cultured mouse and human hepatocytes, PANDER was co‐localized with FOXO1 in the nucleus. PANDER directly interacted with FOXO1 in mouse and human hepatocytes. PANDER overexpression enhanced PANDER‐FOXO1 interaction, and detained FOXO1 in the nucleus upon insulin stimulation in hepatocytes. With the increase in PANDER‐FOXO1 interaction, PANDER overexpression upregulated the expression of gluconeogenic genes and promoted gluconeogenesis in both human and mouse hepatocytes. Luciferase reporter assays further revealed that PANDER augmented the transcriptional activity of FOXO1 on gluconeogenic genes. Moreover, PANDER overexpression also interfered the binding of AS1842856, a specific FOXO1 inhibitor, with FOXO1, and impaired its inhibitory effects on gluconeogenic gene expression and gluconeogenesis in hepatocytes. siRNA mediated‐silencing of FOXO1 inhibited PANDER‐promoted gluconeogenic gene expression and glucose production in hepatocytes. In conclusion, PANDER protein is abundantly present in the nucleus, where it functions as a new co‐activator of FOXO1 to induce gluconeogenic gene expression in hepatocytes.

## 1 INTRODUCTION

Family with sequence similarity 3 (FAM3) cytokine‐like gene superfamily consists four members designated as FAM3A, FAM3B, FAM3C and FAM3D respectively.^1^ In pancreas, FAM3B is highly expressed in the islets,^2^, ^3^ and it is thus also called pancreatic derived factor (PANDER). PANDER is co‐secreted with insulin in pancreatic β cells upon the stimulation of glucose and other insulin secretagogues.^4^ Islet‐secreted PANDER contributed to islet β cell dysfunctions under obese or stress conditions.^5^, ^6^, ^7^, ^8^, ^9^ PANDER also binds to the liver membrane and induces insulin resistance.^10^ Specific overexpression of PANDER in mouse islets causes hepatic insulin resistance, and enhances gluconeogenesis and lipogenesis,^11^, ^12^ further confirming that the liver is one target tissue of pancreatic‐derived PANDER. Moreover, PANDER is also abundantly expressed in the livers of human and rodents, and hepatic PANDER expression is increased under obese condition.^13^ Hepatic PANDER overexpression promotes gluconeogenesis and lipogenesis, whereas hepatic PANDER silencing ameliorated fatty liver and hyperglycemia in obese mice.^13^, ^14^ PANDER knockout (PANKO) mice exhibit increased hepatic insulin sensitivity.^15^, ^16^ In humans, increased circulating PANDER levels have been reported to be associated with pancreatic β cell dysfunction, hyperglycemia and insulin resistance in various races.^17^, ^18^, ^19^ Overall, these clinical and experimental studies had established that PANDER plays important roles in the regulation of glucose and lipid metabolism.^17^, ^18^, ^19^, ^20^

In the previous study,^13^ we demonstrated that PANDER promotes the lipogenesis in hepatocytes by inhibiting Akt to activate FOXO1. However, whether PANDER activates FOXO1 activity via other mechanism(s) beyond Akt repression remains unknown. So far, whether or not PANDER can be released by other non‐islet cell types such as hepatocyte remains unclear. A putative secretory isoform of PANDER had been reported to be detected in the medium of cultured hepatocytes after PANDER overexpression,^14^ while the secretory PANDER isoform failed to be detected in mouse livers and cultured hepatocytes in several other studies.^13^, ^21^, ^22^ Moreover, a non‐secretory PANDER isoform has been reported to promote invasion and metastasis of human colon cancer cells.^23^ Collectively, these findings had raised an important hypothesis that PANDER may modulate glucose and lipid metabolism via non‐secretory mechanism in hepatocytes.

In the current study, we determined that PANDER is abundantly present in the nucleus of both human and mouse hepatocytes. Under diabetic conditions, nuclear distribution of PANDER is increased in the livers. PANDER directly interacts with FOXO1 to activate it in the nucleus, enhancing gluconeogenic gene expression and promoting gluconeogenesis in hepatocytes.

## 2 MATERIALS AND METHODS

### 2.1 Animals

Eight‐week‐old male C57BL/6 mice and 8‐ to 12‐week‐old male db/db and db/m (C57BKS background Jackson Laboratory, USA) were used in this study. 8‐10 week old male C57BL/6 mice were fed on 45% high fat diet (HFD) or normal diet (ND) for 12 weeks to induce diabetic and steatotic phenotype as detailed previously.^13^, ^24^ 12‐ to 16‐ week old male db/db mice and db/m mice on BKS ground were also used in this study.

### 2.2 Immunohistochemistry

Liver tissues were fixed, dehydrated and embedded in paraffin wax. Sections (5‐μm thick) were incubated with anti‐PANDER (1:200, ABclonal, A1082) or IgG as primary antibody overnight at 4°C. Secondary antibodies (Zhongshan Golden Bridge, China) were added for 30 minutes at 37°C. Slides were counterstained with 3,30‐diaminobenzidine (DAB) and hematoxylin.

### 2.3 Mitochondria and cytosol/nuclear fraction isolation

Mitochondria fraction was isolated using Mitochondria Isolation Kit (Applygen Technologies Inc, C1260, China) as detailed previously.^25^ In brief, 100 mg mouse liver tissues were homogenized in Mito Solution by glass homogenizer in the ice. All of procedures were in ice or 4°C. Then the lysate was centrifuged twice at 800 *g* for 5 minutes, then the supernatant was collected and centrifuged at 10 000 *g* for 10 minutes, the mitochondria precipitated at the bottom of the tube, and cytosolic fraction was in the supernatant. 0.2 ml Mito solution was added to the tube and centrifuged at 12 000 *g* for 5 minutes to wash the mitochondria at least three times. Finally, the mitochondria were re‐suspended in 50 μl Roth lysis buffer for immunoblotting.

### 2.4 Nuclear cytosol extraction

Nuclear and cytosolic fractions were isolated using the Nuclear Cytosol Extraction Kit (Applygen Technologies Inc, P1200, China). All processes were carried out on ice and all reagents were pre‐cooled before the experiment. One hundred milligram mouse liver tissues were homogenized in 1 ml Cytosol Extraction Buffer A (CEB‐A) by a glass homogenizer in ice 20‐40 times. The lysate was transferred to a pre‐cooled 1.5 ml centrifuge tube. After oscillating vigorously for 30 seconds, the lysate was kept on ice for 10‐15 minutes, and oscillated for 15 seconds every 5 minutes. Then, 50 μl of Cytosol Extraction Buffer B (CEB‐B) was added, and the mixture was oscillated for 10 seconds. After placing on ice for 1 minute, the lysis was centrifuged at 1000 *g* for 5 minutes at 4°C. The precipitation was a nuclear crude extraction and the supernatant was a crude cytoplasmic protein component. The supernatant was transferred to another pre‐cooled centrifuge tube, and centrifuged at 12 000 *g* at 4°C for 10 minutes. The supernatant is cytosol protein component. 100 μl CEB‐A and 5 μl CEB‐B were added to the raw nuclear extraction and vortexed for 10 seconds, then the mixture was incubated on ice for 1 minute and centrifuged at 1000 *g* for 5 minutes at 4°C, the procedure was repeated again. One hundred microlitre pre‐cooled Nuclear Extraction Buffer (NEB) (with 0.1 μl DTT, 0.5 μl PMSF and 0.5 μl proteinase inhibitor) was added into the precipitate, and the mixture was kept on ice for 30 minutes after 15 seconds of intense oscillation, vortexing for 15 seconds every 10 minutes. The mixture was centrifuged at 12 000 *g* for 5 minutes, and the supernatant obtained was a nuclear protein. Twenty microgram cytosolic or nuclear protein was used for immunoblotting assays.

### 2.5 Primary mouse hepatocytes culture

Primary mouse hepatocytes were cultured as detailed previously.^24^ Briefly, the mouse liver was perfused with 50 mL Kreb solution, followed by 30 mL Kreb solution with collagenase type Ι to digest the liver. The cells were filtered with a metal mesh using 1640 medium; the cells were then washed and centrifuged at 50 g at 4°C for three times. The cells were cultured with 1640 containing 10% FBS for 6‐8 hours at 37°C with 5% CO_2_ before treating.

### 2.6 Cell culture

The human hepatocarcinoma cell line (HepG2) was purchased from the American Type Culture Collection (ATCC) and maintained in high‐glucose solution (25 mmol L^–1^) DMEM (Invitrogen, USA) and 10% foetal bovine serum (FBS). HepG2 cells or mouse hepatocytes were infected with 50 multiplicity of infection (MOI) of adenovirus (Ad‐LacZ or Ad‐PANDER, which expresses wild type and full length mouse PANDER gene^13^). After adenovirus infected for 32 hours, cells were serum starved for 12 hours, then stimulated with 100 nmol L^–1^ insulin (Novo Nordisk) for 30 minutes before immunofluorescent staining. HepG2 cells were infected with Ad‐GFP or Ad‐PANDER for 20 hours, and then treated with or without FOXO1 inhibitor AS1842856 (1 μmol L^–1^, Selleck, China) in FBS‐free DMEM medium for 18 hours.

### 2.7 Glucose production assays

The experimental procedure for glucose production was detailed in previous study.^24^ In brief, HepG2 cells were infected with Ad‐GFP or Ad‐PANDER for 30 hours and then washed by PBS for three times. Cells were incubated in glucose production buffer (glucose and phenol red‐free DMEM medium with 20 mmol L^–1^ sodium lactate and 2 mmol L^–1^ sodium pyruvate) with or without FOXO1 inhibitor AS1842856 (1 μmol L^–1^). After culture for 13 hours, 10 nmol L^–1^ insulin was added to the cells and treated for 3 hours. Cell culture supernatants were collected and centrifuged at 1000 *g* for 5 minutes, the supernatants were used to analyse glucose content using Glucose Assay Kit (Sigma‐Aldrich, GAGO‐20). The glucose content was normalized by the cellular protein content in each sample (µg/mg protein), and then the data were normalized to the control value.

### 2.8 Confocal imaging assay

Liver slides, HepG2 cells or primary mouse hepatocytes were washed with PBS to remove the medium and fixed with 4% paraformaldehyde for 15 minutes, then washed with PBS for three times and permeabilized with 0.05% Triton X‐100/0.5% BSA for 10 minutes. The coverslips were blocked in 1% BSA for 30 minutes and incubated with anti‐PANDER antibodies (1:200, ABclonal, A1082) with or without anti‐FOXO1 antibodies (1:100, Cell Signaling technology, 14952) or IgG at 4°C overnight. After washing with PBS for three times, secondary antibodies (goat anti‐rabbit Alexa Fluor 488 for PANDER, and goat anti‐mice Alexa Fluor 594 for FOXO1) were added to the coverslips and incubated for 1 hour. The nucleus was stained with DAPI for 10 minutes, and 50% glycerol in PBS was used to mount the coverslips on glass slides. Mounted coverslips were imaged and cells were visualized by fluorescence microscopy using Confocal Laser Scanning Microscope.

### 2.9 RNA extraction and real time‐PCR assays

Total RNA was extracted from HepG2 cells by TRIzol reagent (Invitrogen, USA), and cDNA was synthesized by cDNA Synthesis Kit (Thermo, USA). Quantitative real‐time PCR was performed using SYBR Green PCR Master Mix (TOYOBO, Japan). The relative levels of the target gene mRNAs were calculated by 2^−ΔΔCt^ methodology using β‐actin as house‐keeping gene. All the primers for PCR assays were listed in Table S1.

### 2.10 Immunoblotting assays

Total protein was extracted from mouse livers or HepG2 cells using Roth lysis buffer containing proteinase inhibitor cocktail (Applygen Technologies Inc, China) and centrifuged for 10 minutes at 4°C at 12 000 *g* to collected supernatants. 20‐80 μg total protein was separated by 10%‐12% SDS‐PAGE. Primary antibodies were incubated in 5% milk at 4°C overnight. Proteins examined in this study include PANDER (ABclonal, A13592), FOXO1 (Cell Signaling technology, 2880S), phosphorylated FOXO1 (Ser256, Cell Signaling technology, 9461S), PEPCK (Bioworld, BS6870), G6Pase (Santa Cruz, sc‐25840), COX 4 (Huaxingbio, HX1842), Lamin B1 (Huaxingbio, HX‐1846), β‐actin (Zhongshan Golden Bridge, TA‐09). ImageJ (version 1.42) was used to analyse protein expression, and data were normalised to β‐actin protein expression.

### 2.11 Immunoprecipitation

Five hundred microgram total protein of mouse livers or HepG2 cells was added in 20 μl Protein A Sepharose CL‐4B beads at 4°C for 1 hour to pre‐clear proteins. Then the samples were centrifuged at 13 000 *g* at 4°C for 1 minute, and then the supernatant was transferred to a new tube. 10 μl (200 μg/ml) anti‐ PANDER (Biosen Biotechnology Co., Beijing, China) or IgG was added into the tube, and then the samples were incubated for 2 hours at 4°C on a Nutator. After incubation, 40 μl of Protein A bead suspension was added in and samples were incubated at 4°C on a Nutator overnight. The samples were centrifuged at 13 000 *g* for 1 minute, and the pellet was washed twice with 0.5 mL buffer I (1% Triton X‐100, 0.1% SDS, 50 mmol L^–1^ HEPES and 150 mmol L^–1^ NaCl, pH 7.8) and buffer II (1% Triton X‐100, 0.1% SDS and 50 mmol L^–1^ HEPES, pH 7.8) respectively. 50 μl of 5X loading buffer containing 0.2 mmol L^–1^ DTT was added. The samples were mixed and boiled at 100°C for 10 minutes, and then centrifuged at 13 000 *g* for 2 minutes. Twenty microlitres of the supernatant was separated by 10% sodium dodecyl sulphate (SDS) gel, and immunoblotting assays were then performed.

### 2.12 Luciferase reporter assay

The fragment of mouse G6pase gene promoter flanking −1360 bp to +2 bp which contains four FOXO1 binding sites^26^ was cloned into the pGL4.11(441‐2)‐basic vector and kindly provided by Prof. Weiping Zhang (Department of Pathophysiology, Second Military Medical University, China). The protocol for promoter activity assay was detailed previously.^24^ The pG6pase promoter‐firefly luciferase and pRL‐TK‐Renilla luciferase were cotransfected with FOXO1 plasmid or FOXO1 and PANDER plasmid or GFP plasmid into HepG2 cells using VigoFect transfection reagent (Vigorous Biotechnology). FOXO1 plasmid was kindly provided by Prof. Ying Zhao of Peking University Health Science Center. The wild type and mutant PANDER plasmids were constructed previously.^4^ After 12 hours, the culture supernatant was replaced with fresh medium. The activities of firefly luciferase and Renilla luciferase were measured with the Dual‐Luciferase reporter assay kit (Promega) at 24 hours post‐transfection. The data in each read were normalised by the data of corresponding Renilla luciferase. Finally, the data were normalised with the control values.

### 2.13 siRNA inhibition of PANDER and FOXO1 expression

For PANDER or FOXO1 knockdown, HepG2 cells were transfected with 50 nmol L^–1^ siRNA mixtures against human PANDER or FOXO1 mRNA (scrambled siRNAs as negative control, Beijing Biolino Nucleic Acid Technology Co., Ltd) for 12 hours. At 12 hours post‐transfection, the medium was replaced with fresh medium, and cells were infected with 50 MOI of Ad‐GFP, Ad‐FOXO1 or Ad‐PANDER for 24 hours before glucose production was determined. All the siRNA sequences were listed in Table S2.

### 2.14 Statistical analysis

Data were displayed as the mean ± SEM, and statistical differences between two groups were analysed by *t* tests. *P* < 0.05 was considered statistically significant.

## 3 RESULTS

### 3.1 PANDER is abundantly expressed in the nucleus of mouse and human hepatocytes

**Figure 1:**
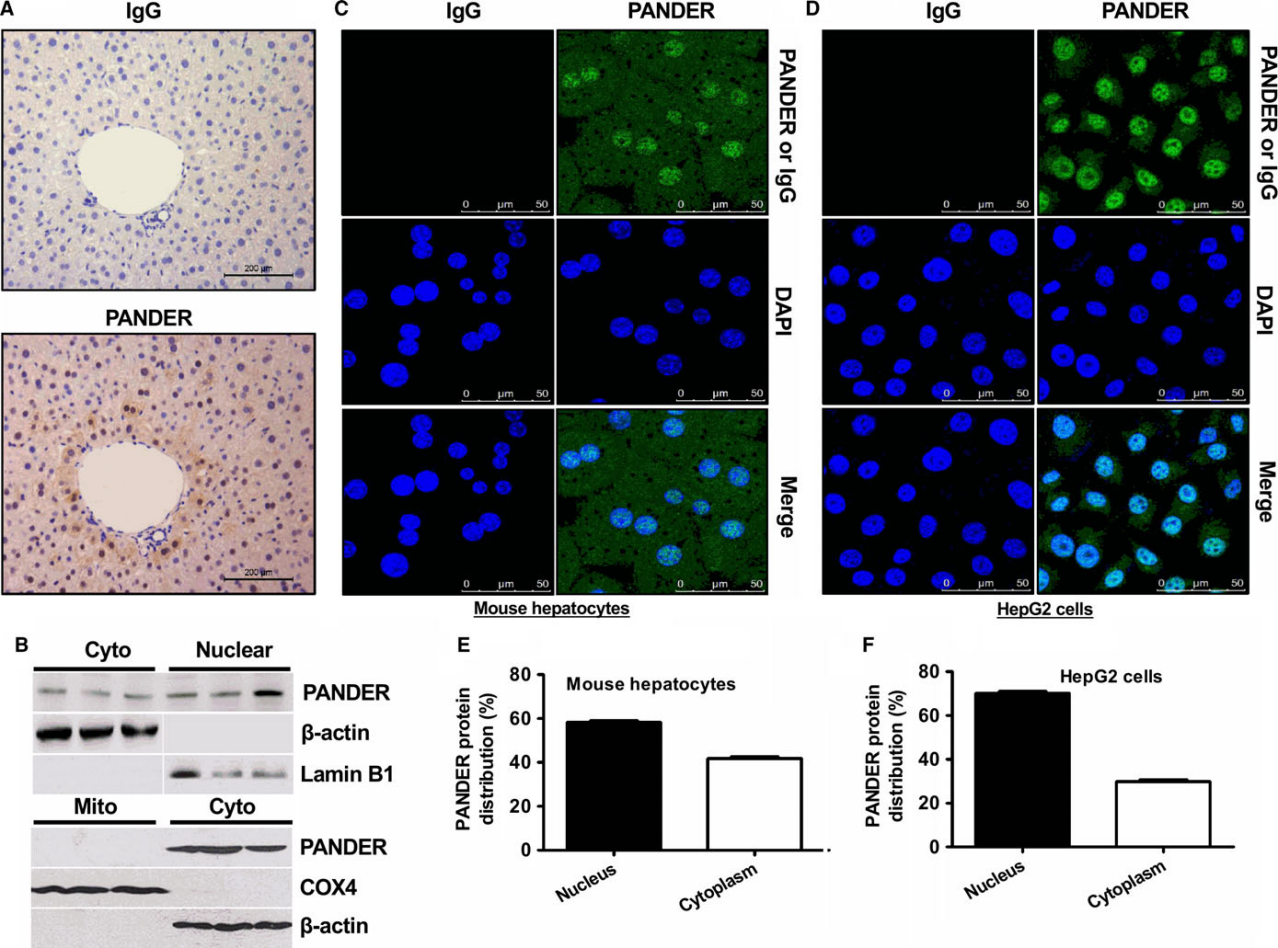
PANDER protein is present in the nucleus of hepatocytes. A, Immunohistochemical staining revealed that PANDER protein is present in both the cytoplasm and nucleus of normal mouse livers. B, PANDER protein is located in nuclear but not mitochondrial fraction of mouse livers. Mitochondrial, cytosolic and nuclear fractions were isolated as detailed in the experimental procedure. Mito, mitochondrial fraction; Cyto, cytosolic fraction; Nuclear, nuclear fraction. COX4, cytochrome c oxidase subunit 4, was used as a mitochondrial biomarker. Lamin B1 was used as a biomarker for nuclear protein. N = 5. (C‐D) Confocal imaging revealed that PANDER protein is abundantly present in the nucleus of primary mouse hepatocytes (C) and human HepG2 cells (D). The images were the representatives of 3 independent experiments. (E‐F) Quantification of PANDER distribution in nucleus and cytoplasm of mouse hepatocytes (E) and HepG2 cells (F) as evaluated by confocal images. At least 50 cells were analysed by confocal imaging system

To probe the mechanism of liver‐derived pancreatic derived factor (PANDER) in regulation of hepatic glucose and lipid metabolism in hepatocytes, its subcellular distribution was determined by immunohistochemical (IHC) staining and confocal imaging analyses in mouse livers and cultured hepatocytes. In mouse livers, IHC staining revealed that PANDER protein is present in both the cytoplasm and nucleus (Figure 1A). Confocal imaging further confirmed the presence of PANDER protein in both the cytoplasm and nucleus of mouse livers (Figure S1). To further validate the subcellular distribution of PANDER protein, mitochondrial, cytosolic and nuclear fractions were isolated respectively. As a result, PANDER protein is present in both cytosolic and nuclear fractions, but not in mitochondrial fraction of mouse livers (Figure 1B). PANDER protein detected in the current study is a full length isoform with the molecular weight of 26kD. Generally, the secretory PANDER isoform (22kD) in mouse livers and cultured hepatocytes cannot be effectively detected. Confocal imaging analyses further confirmed that PANDER protein is abundantly present in the nucleus of primary mouse hepatocytes (Figure 1C) and human HepG2 cells (Figure 1D). Quantitative assays further showed that about 50%‐60% of PANDER protein is distributed in the nucleus of primary mouse hepatocytes (Figure 1E), whereas about 70% of it is present in the nucleus of human HepG2 cells (Figure 1F). Overall, these findings clearly indicated that PANDER protein is abundantly present in the nucleus of both human and mouse hepatocytes.

### 3.2 Nuclear PANDER distribution is increased in the livers of obese diabetic mice

**Figure 2:**
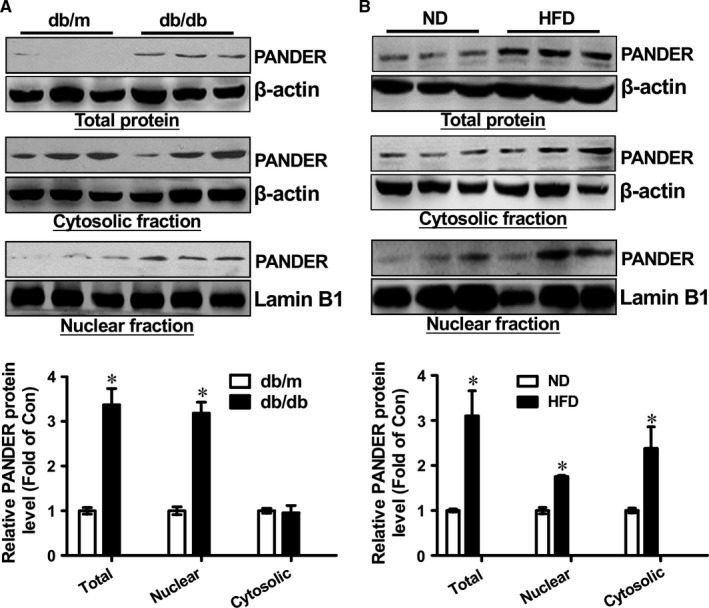
Nuclear PANDER distribution is increased in the livers of obese diabetic mice. A, Nuclear expression of PANDER is increased in the livers of db/db mice. Cytosolic and nuclear fractions of mice were isolated for PANDER expression analyses. The representative gel images were shown in upper panel, and quantitative data shown in lower panel. B, Nuclear expression of PANDER is increased in the livers of HFD mice. C57BL/6 mice were fed on high fat diet (HFD) for 12 wk. PANDER protein in the cytosolic and nuclear fractions was analysed by western blotting assays. The representative gel images were shown in upper panel, and quantitative data shown in lower panel. ND, mice fed on normal diet; HFD, mice fed on high fat diet. N = 3‐5, *P < 0.05 vs control mice (db/m or ND mice)

Abundant distribution of PANDER in the nucleus suggested that it may impact hepatic glucose metabolism via the intracellular mechanism beyond secretion. To determine the role of nuclear PANDER in the pathogenesis of diabetes, its expression was analysed in the livers of obese diabetic mice. As a result, total PANDER expression was increased in db/db mouse livers (Figure 2A), which is consistent with the previous findings.^13^ Determination of subcellular PANDER content revealed that nuclear PANDER distribution was significantly increased, whereas its cytosolic distribution remained unchanged in db/db mouse livers when compared with db/m mouse livers (Figure 2A). In high fat diet (HFD)‐fed obese mouse livers, the total PANDER expression was also increased when compared with normal diet (ND)‐fed mouse livers. Furthermore, both nuclear and cytosolic PANDER distributions were increased in HFD mouse livers when compared with normal mouse livers (Figure 2B). Overall, these findings revealed that nuclear PANDER distribution was increased in the mouse livers under obese condition.

### 3.3 PANDER interacts with FOXO1 in mouse and human hepatocytes

**Figure 3:**
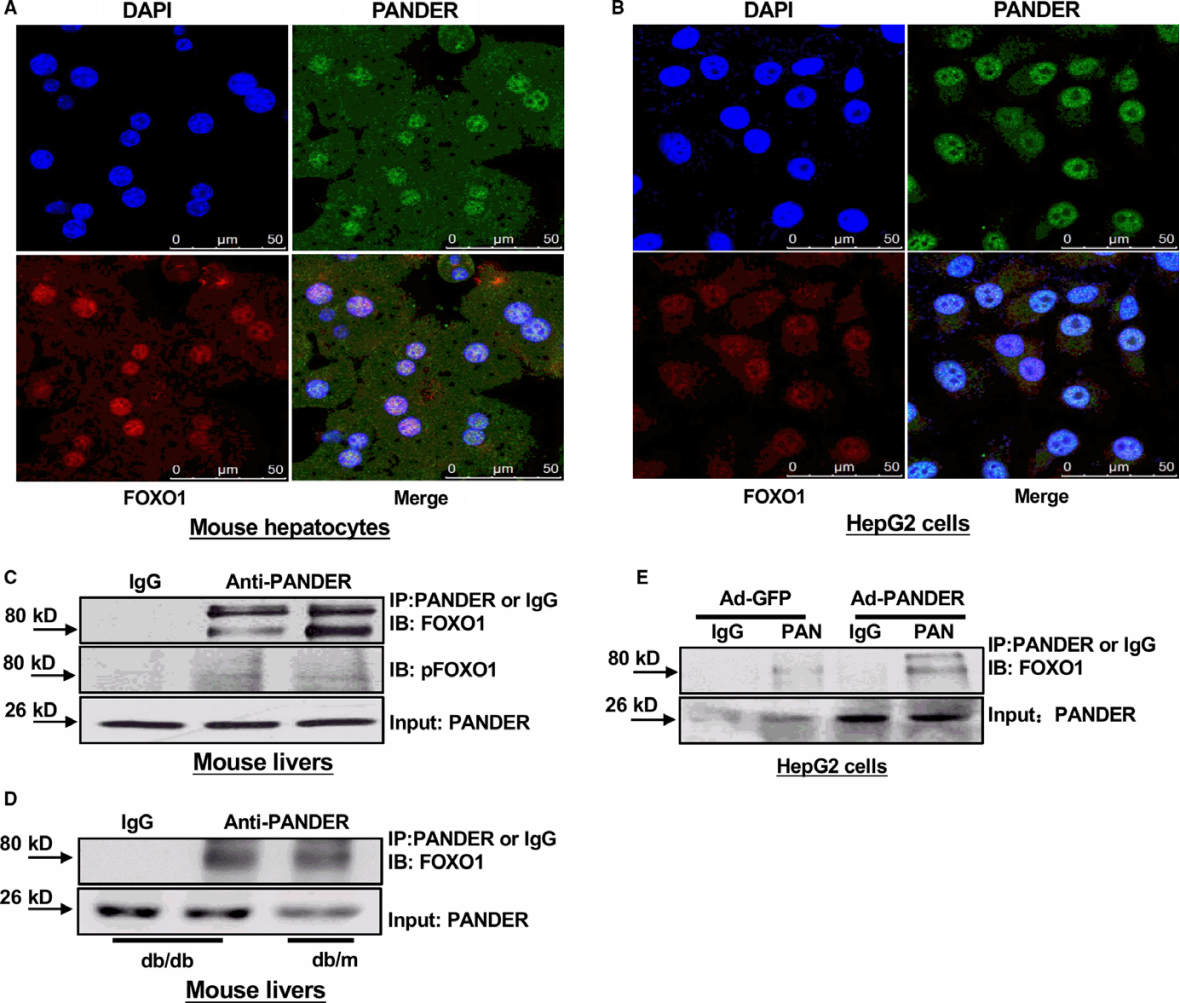
PANDER interacts with FOXO1 in mouse livers and hepatocytes. (A‐B) Confocal imaging revealed that PANDER and FOXO1 are co‐localized in the nucleus of mouse hepatocytes (A) and human HepG2 cells (B). The images were the representatives of 3 independent experiments. C, PANDER interacted with FOXO1 in mouse livers. D, PANDER‐FOXO1 interaction was increased in the livers of db/db mice when compared with db/m mice. E, PANDER interacted with FOXO1 in human HepG2 cells. Cells were infected with Ad‐GFP or Ad‐PANDER for 24 h before Co‐IP was performed. Co‐IP was performed using mouse liver or cell lysate as detailed in the methodology

**Figure 4:**
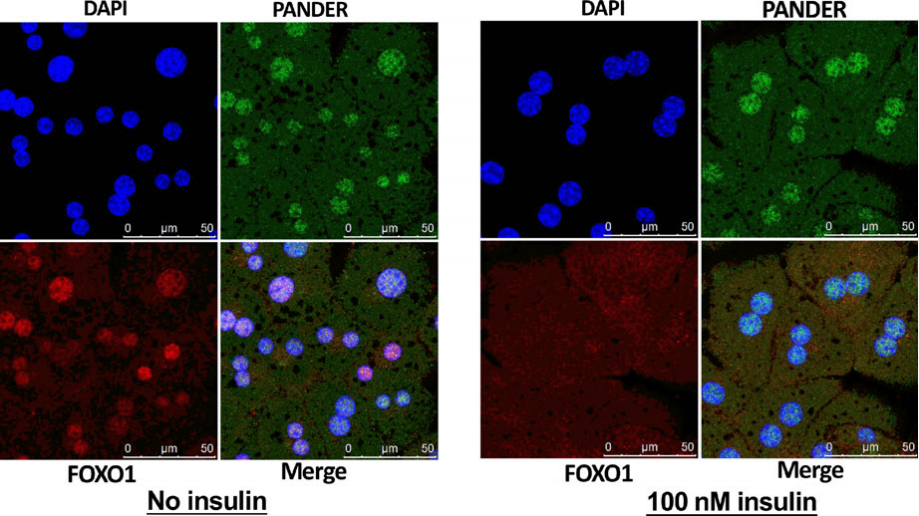
Insulin stimulated the nuclear exclusion of FOXO1 but not PANDER in mouse hepatocytes. Mouse hepatocytes were stimulated with 0 or 100 nmol L^–1^ insulin for 30 min before being performed for confocal imaging. The images were the representatives of 3 independent experiments

**Figure 5:**
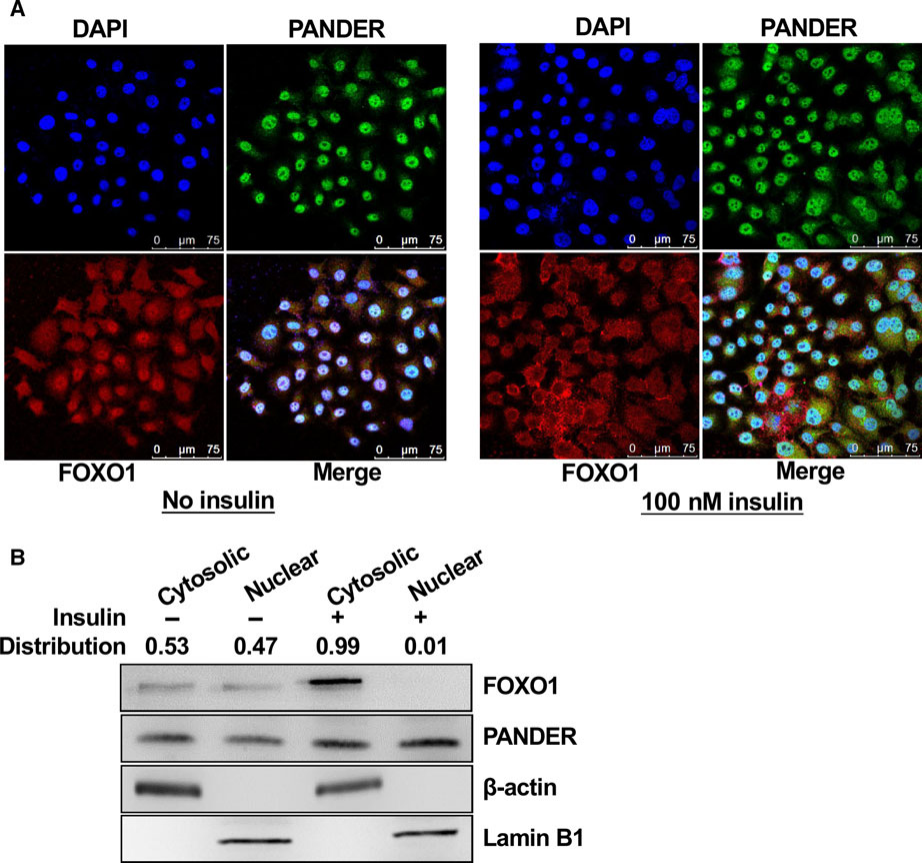
Insulin stimulated the nuclear exclusion of FOXO1 but not PANDER in HepG2 cells. A, Cells were stimulated with 0 or 100 nmol L^–1^ insulin for 30 min before being performed for confocal imaging. The images were the representatives of 3 independent experiments. B, FOXO1 and PANDER distribution in cytosolic and nuclear fractions of HepG2 cells stimulated with or without insulin for 30 min. The images were the representatives of 3 independent experiments, and the average distribution values of FOXO1 in nuclear and cytosolic fractions in the absence or presence of insulin stimulation were presented as numbers. β‐actin and Lamin B1 were used as a biomarker for cytosolic and nuclear proteins, respectively

Because both PANDER and FOXO1 distributions were increased in the livers under diabetic conditions, and PANDER overexpression or silencing increased or decreased FOXO1 protein level in mouse livers,^13^, ^27^ whether a direct interaction existed between them was further determined. Firstly, confocal imaging revealed that PANDER is co‐localized with FOXO1 in the nucleus of primary mouse hepatocytes (Figure 3A). Similarly, PANDER is also co‐localized with FOXO1 in the nucleus of HepG2 cells (Figure 3B). Thus, co‐immunoprecipitation (Co‐IP) was further performed to validate whether PANDER directly interacted with FOXO1 in mouse lives and cultured hepatocytes. The results indicated that PANDER interacted with FOXO1 and phosphorylated FOXO1 in mouse livers (Figure 3C). In db/db mouse livers, with the increase of PANDER expression, PANDER‐FOXO1 interaction was increased when compared with that in db/m mouse livers (Figure 3D). In cultured HepG2 cells, PANDER also interacted with FOXO1, and their interaction was enhanced after PANDER overexpression (Figure 3E). Generally, insulin activates Akt to phosphorylate FOXO1 and promote its nuclear exclusion. To determine the change in PANDER‐FOXO1 co‐localization upon insulin simulation, cytosolic and nuclear PANDER and FOXO1 distributions in the absence or presence of 100 nmol L^–1^ insulin stimulation were analysed in mouse hepatocytes and HepG2 cells. In the absence of insulin stimulation, both PANDER and FOXO1 were predominantly co‐localization in the nucleus of primary mouse hepatocytes (Figure 4, left panel). Upon insulin stimulation, PANDER was still localised in the nucleus, whereas most of the FOXO1 was translocated to the cytoplasm (Figure 4, right panel) in mouse hepatocytes. Insulin similarly stimulated the nuclear exclusion of FOXO1, but not the PANDER in human HepG2 cells (Figure 5A). In support, subcellular FOXO1 and PANDER distribution analyses confirmed that FOXO1 but not PANDER was translocated to cytoplasm upon insulin stimulation in HepG2 cells (Figure 5B). Overall, these findings revealed that acute insulin stimulation failed to affect PANDER distribution in the nucleus in hepatocytes.

### 3.4 PANDER co‐activates FOXO1 to upregulate gluconeogenic genes and promote gluconeogenesis in hepatocytes

**Figure 6:**
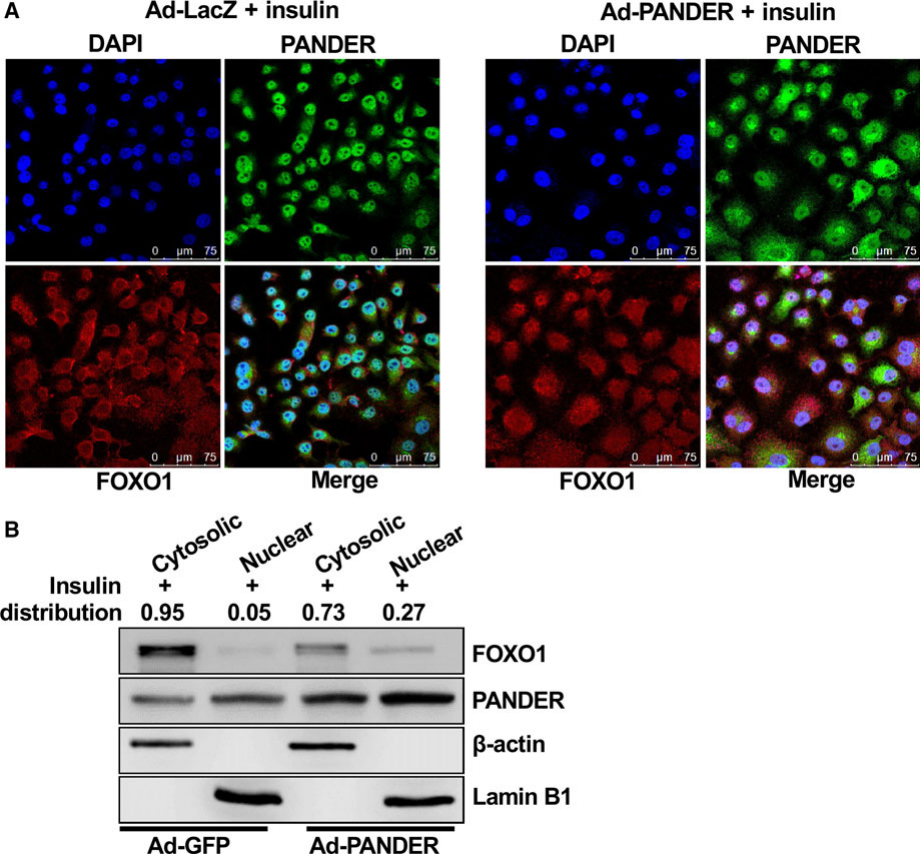
PANDER overexpression detained FOXO1 in the nucleus upon insulin stimulation in HepG2 cells. A, Cells infected with Ad‐LacZ or Ad‐PANDER for 32 hours and starved for 12 hours, and then stimulated with 100 nmol L^–1^ insulin for 30 min before being performed for confocal imaging. The images were the representatives of 3 independent experiments. B, FOXO1 and PANDER distribution in cytosolic and nuclear fractions of HepG2 cells stimulated with insulin for 30 min. The images were the representatives of 3 independent experiments, and the average distribution values of FOXO1 in nuclear and cytosolic fractions in the presence of insulin stimulation were presented as numbers. β‐actin and Lamin B1 were used as a biomarker for cytosolic and nuclear proteins, respectively

**Figure 7:**
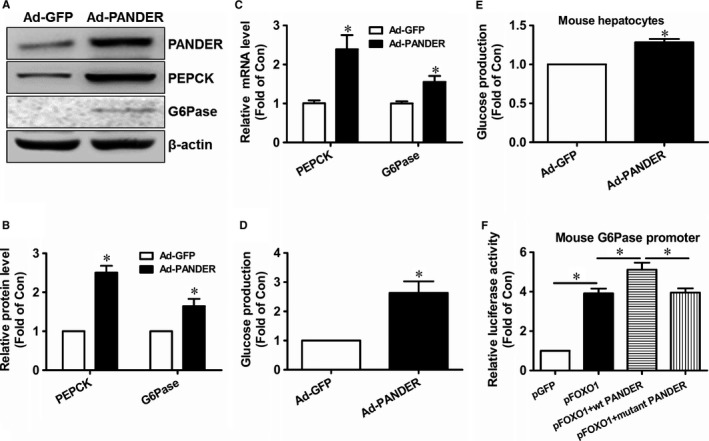
PANDER promoted gluconeogenesis in HepG2 cells. (A‐B) PANDER overexpression upregulated the protein levels of gluconeogenic genes PEPCK and G6Pase. Representative gel images were shown panel A, and quantitative data shown in panel B. C, PANDER overexpression upregulated the mRNA levels of gluconeogenic genes PEPCK and G6Pase. D, PANDER overexpression promoted gluconeogenesis in HepG2 cells. E, PANDER overexpression promoted gluconeogenesis in primary mouse hepatocytes. F, PANDER augmented FOXO1’s activation on the promoter activity of mouse G6Pase gene. N = 3‐5, *P < 0.05 vs control cells, or between two indicated groups of cells

FOXO1 plays a decisive role in regulating gluconeogenesis by controlling the expression of two key gluconeogenic genes, PEPCK and G6Pase, in hepatocytes.^28^ Because PANDER overexpression increased PANDER‐FOXO1 interaction, whether it increased FOXO1 activity was evaluated by analysing its impact on FOXO1 distribution and the expression of FOXO1 target genes. In Ad‐LacZ‐infected HepG2 cells, insulin markedly promoted the nuclear exclusion of FOXO1. However, insulin‐stimulated FOXO1 nuclear exclusion is impaired in Ad‐PANDER‐infected HepG2 cells (Figure 6A). In support, subcellular FOXO1 and PANDER distribution analyses confirmed that PANDER overexpression detained more FOXO1 in the nucleus (Nuclear/cytosolic fraction ratio is 0.27/0.73 in Ad‐PANDER‐treated cells) of HepG2 cells upon insulin stimulation when compared with control cells (Nuclear/cytosolic fraction ratio is 0.05/0.95 in Ad‐GFP‐treated cells) (Figure 6B). Overall, these findings suggested that PANDER overexpression repressed insulin‐stimulated nuclear exclusion of FOXO1. To further validate whether PANDER overexpression increased FOXO1 activity, its impact on gluconeogenic gene expression and gluconeogenesis was determined. In HepG2 cells, PANDER overexpression upregulated the mRNA and protein levels of PEPCK and G6Pase (Figure 7A‐C). Consistently, PANDER overexpression stimulated gluconeogenesis in HepG2 cells (Figure 7D). PANDER overexpression similarly stimulated glucose production in primary mouse hepatocytes (Figure 7E). Then, whether PANDER directly stimulated the transcriptional activity of FOXO1 was further evaluated using luciferase reporter driven by mouse G6pase gene promoter. As a result, FOXO1 significantly activated the promoter activity of mouse G6pase gene, and PANDER overexpression augmented the activation (Figure 7F). In contrast, a mutant PANDER with the mutations of Cys91 and Cys229, which form one disulphide bond, to serines, failed to augment FOXO1 transcriptional activity (Figure 7F). The mutant PANDER cannot be processed and released from pancreatic β cells due to the change of structure.^4^

**Figure 8:**
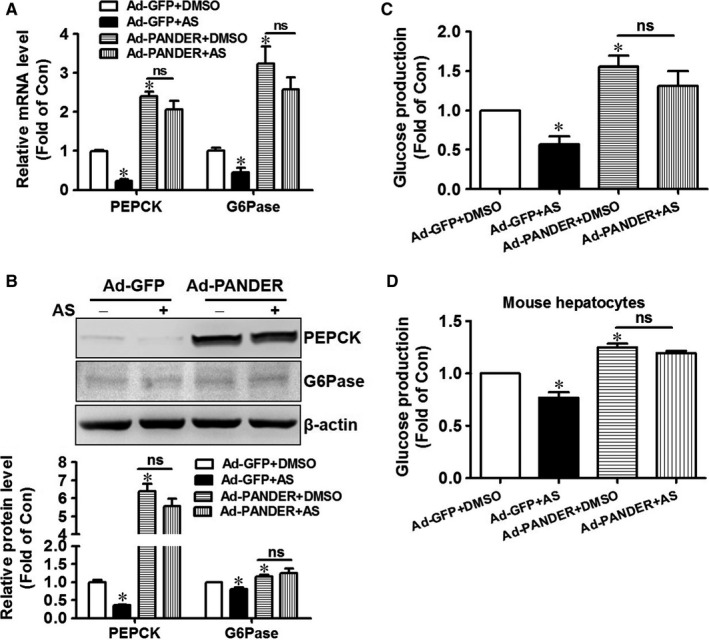
AS1842856 failed to repress FOXO1 activity and glucose production in hepatocytes after PANDER overexpression. A, AS1842856 treatment on the mRNA levels of gluconeogenic genes in Ad‐PANDER‐infected HepG2 cells. B, AS1842856 on the protein levels of gluconeogenic genes in Ad‐PANDER‐infected HepG2 cells. The representative gel images were shown in upper panel, and quantitative data shown in lower panel. C, AS1842856 failed to suppress gluconeogenesis in Ad‐PANDER infected HepG2 cells. D, AS1842856 failed to significantly suppress gluconeogenesis in Ad‐PANDER‐infected mouse hepatocytes. AS, FOXO1 inhibitor AS1842856; Ad‐GFP+AS, Ad‐GFP‐infected cells were treated with AS1842856; Ad‐PANDER+AS, Ad‐PANDER‐infected cells were treated with AS1842856. NS, no significant difference. N = 3‐4, *P < 0.05 vs control cells infected with Ad‐GFP

To further confirm that PANDER‐FOXO1 interaction increase the binding of FOXO1 to its target genes, AS1842856, a specific inhibitor of FOXO1, was used in further study. AS1842856 treatment significantly reduced the mRNA levels of PEPCK and G6Pase in Ad‐GFP‐infected HepG2 cells. However, it failed to significantly reduce them in Ad‐PANDER‐infected cells (Figure 8A). Similarly, AS1842856 treatment reduced the protein levels of PEPCK and G6Pase in Ad‐GFP‐infected cells, but not in Ad‐PANDER‐infected cells (Figure 8B). Consistent with the changes in gluconeogenic gene expression, AS1842856 suppressed gluconeogenesis in control cells, but not in cells after PANDER overexpression (Figure 8C). In primary mouse hepatocytes, AS1842856 also suppressed glucose production in Ad‐GFP‐infected cells but not in Ad‐PANDER‐infected cells (Figure 8D).

**Figure 9:**
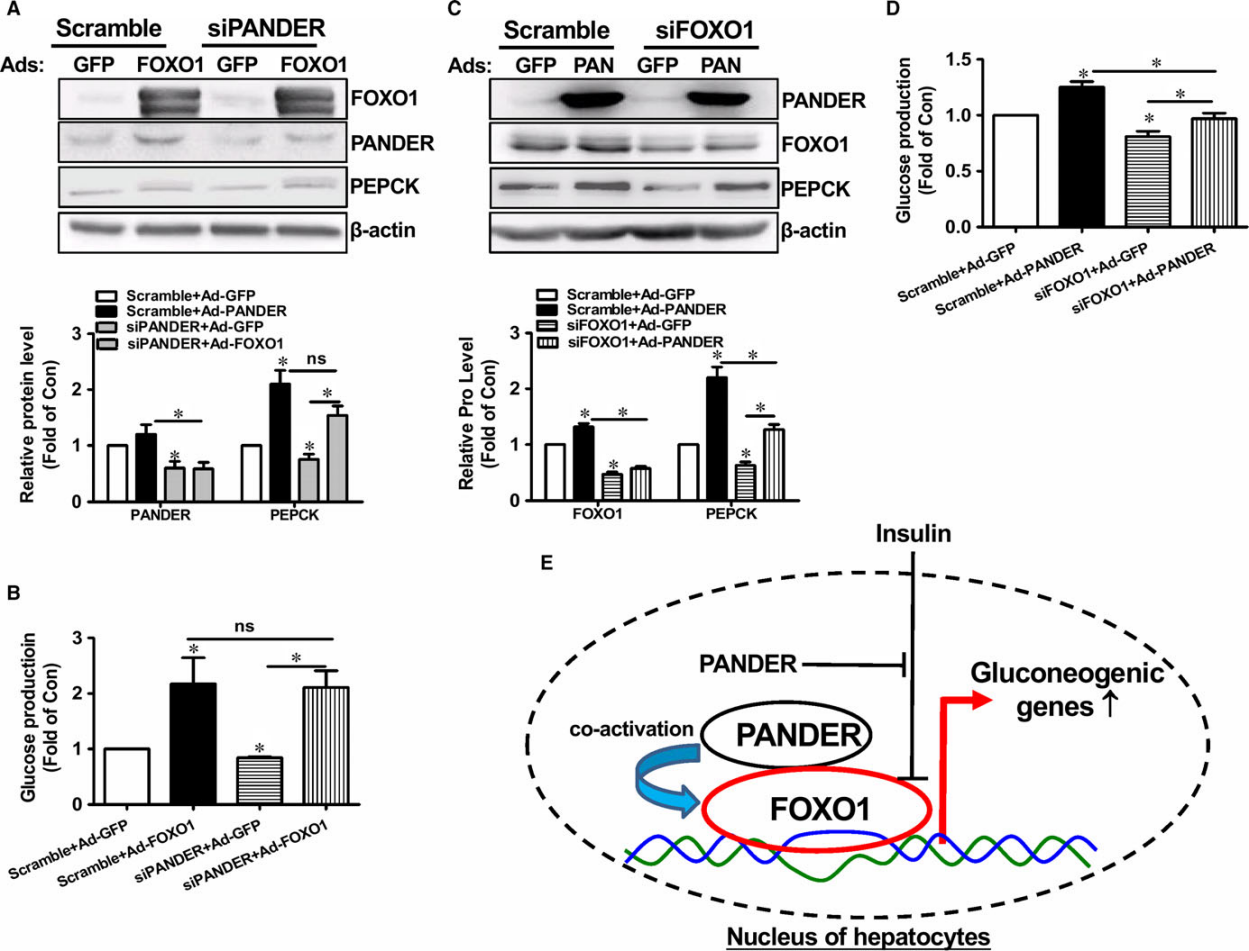
Silencing of FOXO1 inhibited PANDER‐promoted glucose production in HepG2 cells. (A‐B) Silencing of PANDER had little effect on FOXO1‐induced gluconeogenic gene expression (A) and glucose production (B) in HepG2 cells. (C‐D) Silencing of FOXO1 inhibited PANDER‐induced gluconeogenic gene expression (C) and glucose production (D) in HepG2 cells. N = 3‐5, *P < 0.05 vs control cells, or between two indicated groups of cells. E, Proposed model of hepatic PANDER in regulating FOXO1 activity. In the nucleus of hepatocytes, PANDER functions as a co‐activator of FOXO1 to induce the expression of gluconeogenic genes. Under obese condition, an increase in PANDER expression results in FOXO1 activation and prevents insulin‐mediated FOXO1 nuclear exclusion

To further determine that PANDER activated FOXO1 to promote gluconeogenesis, PANDER and FOXO1 expressions were knockdown by siRNA transfection. Silencing efficacy evaluation indicated that siRNA transfection reduced the PANDER and FOXO1 mRNA levels by about 40%‐50% in HepG2 cells (Figure S2A‐B). Silencing of PANDER reduced gluconeogenic gene expression and glucose production in Ad‐GFP‐infected HepG2 cells (Figure 9A‐B), further confirming that PANDER promoted gluconeogenesis in hepatocytes. However, PANDER silencing failed to affect FOXO1‐induced gluconeogenic gene expression and glucose production (Figure 9A‐B). Silencing of FOXO1 inhibited gluconeogenic gene expression and glucose production in HepG2 cells in Ad‐GFP‐infected cells (Figure 9C‐D). Moreover, FOXO1 silencing inhibited PANDER‐induced gluconeogenic gene expression and glucose production in HepG2 cells (Figure 9C‐D).

## 4 DISCUSSION

In a Chinese population, circulating PANDER is increased in patients with metabolic syndrome. In particular, circulating PANDER can predict the risk of type 2 diabetes in Chinese population.^17^ Another clinical report confirmed that serum PANDER level is increased in patients with metabolic syndrome, and an increase in circulating PANDER level can predict metabolic syndrome in Chinese population.^29^ An increase in serum PANDER levels have been reported to be associated with pancreatic β cell dysfunction and hyperglycemia in other races.^18^, ^19^ Overall, these important clinical studies revealed that circulating PANDER is a unique diagnostic biomarker for diabetes and metabolic syndrome, and it is necessary to further study the mechanism(s) of PANDER in the regulation of glucose and lipid metabolism.

In the current study, we demonstrated that PANDER protein is abundantly present in the nucleus of both human and mouse hepatocytes. Under diabetic conditions, nuclear PANDER distribution was increased in the livers. PANDER was further shown to interact with FOXO1, one of the key transcription factors controlling gluconeogenic gene expression and gluconeogenesis. Generally, insulin activates Akt through insulin receptor or insulin receptor substrates pathway to phosphorylate FOXO1. Phosphorylated FOXO1 transfers from nucleus to the cytoplasm, resulting in decreased gluconeogenic gene expression. So far, Akt‐mediated phosphorylation of FOXO1 is the main mechanism for its activity repression.^30^ In addition, cyclin‐dependent kinase 2 (CDK2) and MAPK/ERK signalling pathways also phosphorylate FOXO1 to inhibit it.^31^ In oxidative stress, CREB binding protein (CBP) acetylates the lysine residues in the DNA binding area of FOXO1, interfering its binding with the promoters of target genes.^32^ In our previous study, we had demonstrated that PANDER overexpression elevated FOXO1 protein level with Akt repression, whereas PANDER inhibition reduced FOXO1 protein level with Akt activation in mouse livers and cultured hepatocytes.^13^

The current study further revealed that PANDER activates FOXO1 activity via a direct interaction. PANDER overexpression enhances PANDER‐FOXO1 interaction, activates FOXO1 to induce gluconeogenic gene expression and promote gluconeogenesis in hepatocytes. Moreover, silencing of FOXO1 impaired PANDER‐promoted gluconeogenesis in hepatocytes, while silencing of PANDER had little effect on FOXO1‐triggered glucose production in hepatocytes. These findings together suggested that PANDER promoted gluconeogenesis mainly by activating FOXO1. Interesting, PANDER overexpression repressed the ability of FOXO1 inhibitor AS1842856 to repress gluconeogenic gene expression and gluconeogenesis in both the human and mouse hepatocytes. AS1842856 class of inhibitors directly binds to FOXO1 and prevents its binding with the promoter regions of gluconeogenic genes including PEPCK and G6Pase, repressing gluconeogenic gene expression and gluconeogenesis in cultured hepatocytes and diabetic mouse livers.^33^, ^34^ AS1842856 had no effect on FOXO1 phosphorylation^33^, ^34^ and FOXO1 nuclear exclusion (data not shown), which is different from Akt‐, CDK2‐ and ERK‐mediated FOXO1 phosphorylation and repression. Moreover, AS1842856 does not bind to phosphorylated FOXO1.^33^, ^34^ The EC50 of AS1842856 on inhibiting FOXO1 activity is about 0.03 µmol L^–1^.^33^ In our study, 1 µ mol L^–1^ AS1842856 was used. The results revealed that high concentration of AS1842856 failed to block FOXO1 activity in case of PANDER overexpression by 2‐3 folds. It is likely that PANDER overexpression‐triggered increase in PANDER‐FOXO1 interaction induced FOXO1 structure change and interfered the binding of AS1842856 with FOXO1. Furthermore, an increase in PANDER‐FOXO1 interaction also prevents insulin‐mediated nuclear exclusion of FOXO1. Failure of insulin to suppress hepatic FOXO1 activity and gluconeogenesis is one of the key characteristics of insulin resistance in the liver.^35^ Under obese condition, nuclear PANDER distribution was increased by about 2‐3 folds, which enhanced PANDER‐FOXO1 interaction. Clearly, an increase in PANDER‐FOXO1 interaction is a new mechanism for promoting hepatic gluconeogenesis and subsequent insulin resistance. These findings suggested that PANDER interacts with FOXO1 to detain it in the nucleus, and activate it. PANDER‐FOXO1 interaction provides a novel mode for modulating the activity of FOXO1 beyond Akt‐ or other kinase‐mediated phosphorylation. Under obese condition, an increase in nuclear PANDER distribution enhances PANDER‐FOXO1 interaction, preventing insulin‐mediated FOXO1 nuclear translocation and finally enhancing gluconeogenesis in the liver. In case of FOXO1 inhibition by siRNA, PANDER overexpression still induced gluconeogenic gene expression. Given that siRNA transfection reduced FOXO1 protein level by about 50%, it is likely that PANDER overexpression activated the activity of residual FOXO1. However, the possibility that PANDER also induced gluconeogenic gene expression by other pathway(s) beyond FOXO1 cannot be precluded. For example, PANDER overexpression may activate FOXO3 and FOXO6,^36^, ^37^, ^38^ which can be phosphorylated and inactivated by Akt, to induce gluconeogenic gene expression by repressing Akt in hepatocytes.^13^ Collectively, the current and previous findings revealed that PANDER regulates hepatic FOXO1 activity by inducing Akt repression^13^ and functioning as a direct co‐activator. We and others had demonstrated that FOXO1 overexpression induces the expression of both gluconeogenic and lipogenic genes in mouse livers.^13^, ^39^ Given that overactivation of FOXO1 plays critical roles in triggering hepatic gluconeogenesis and lipogenesis,^27^, ^30^, ^39^, ^40^ targeting PANDER‐FOXO1 interaction will be beneficial for diabetes and fatty liver.

So far, the region in PANDER protein which directly interacts with FOXO1 still remains unknown. Mapping the binding region of PANDER protein with FOXO1 will be helpful for further understanding PANDER‐FOXO1 interaction and FOXO1 activity regulation. To develop PANDER, inhibitors may hold great promise for treating type 2 diabetes and fatty liver. Moreover, PANDER protein has secretory signal peptide, but not typical nuclear location peptide.^1^ So far, how PANDER is translocated into the nucleus in physiological and pathophysiological remains unclear. It is of interest whether PANDER is translocated into the nucleus via the interaction with FOXO1. Another issue is that which cell type(s) could secrete PANDER beyond pancreatic islets cells. Although hepatocyte may not secrete PANDER, an increase in hepatic PANDER expression will trigger gluconeogenesis to cause hyperglycemia, which stimulates PANDER expression and secretion from pancreatic β cells.^4^, ^20^ An increase in circulating PANDER levels will in return exaggerate insulin resistance and induce PANDER expression in hepatocytes.

In summary, PANDER protein is abundantly present in the nucleus of hepatocytes, where it functions as a novel co‐activator of FOXO1. Under obese condition, an increase in nuclear PANDER‐FOXO1 interaction detains FOXO1 in the nucleus, leading to increased gluconeogenesis in hepatocytes (Figure 9E).

## CONFLICT OF INTEREST

The authors declare that there is no conflict of interest.

## AUTHORS’ CONTRIBUTIONS

YC, YM and JW researched data and contributed to the discussion. WY, ZW, ML, DW, LT and BG provided the technical assistance. BG assisted the preparation of manuscript. YC and YM wrote the manuscript. WZ provided the G6pase gene promoter plasmid, and contributed to the discussion. LT provided Ad‐FOXO1 viruses and contributed to the discussion. YC, YM and JY designed the study, and revised/edited manuscript. All authors read and approved the manuscript.
