# Supplementary figures and images for: FAM3B (PANDER) functions as a co‐activator of FOXO1 to promote gluconeogenesis in hepatocytes
Source: J Cell Mol Med. 2018 Nov 28;23(3):1746–58. doi: 10.1111/jcmm.14073 (PMC6378191; doi:10.1111/jcmm.14073)

Suppl figure 1

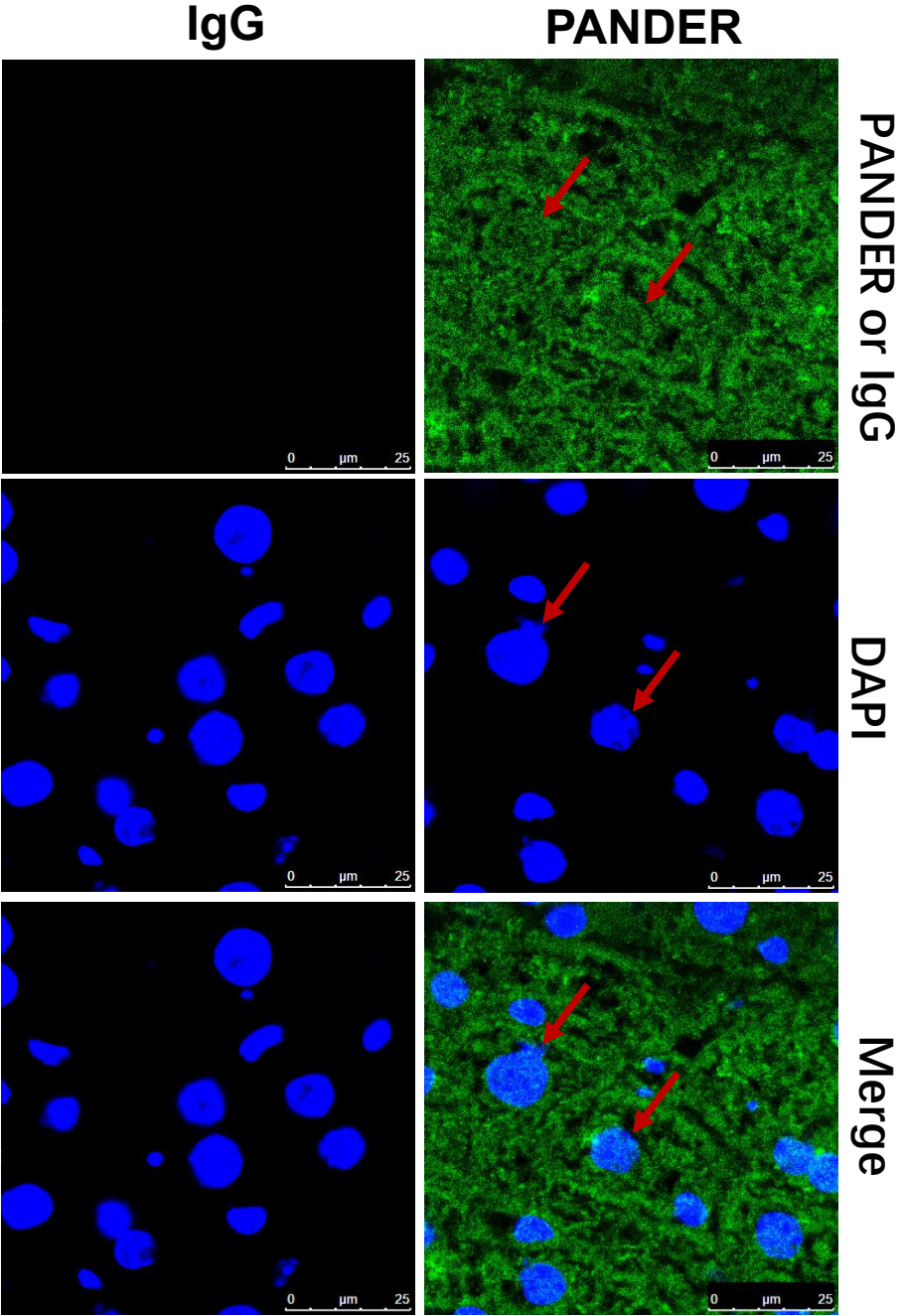

Suppl figure 2

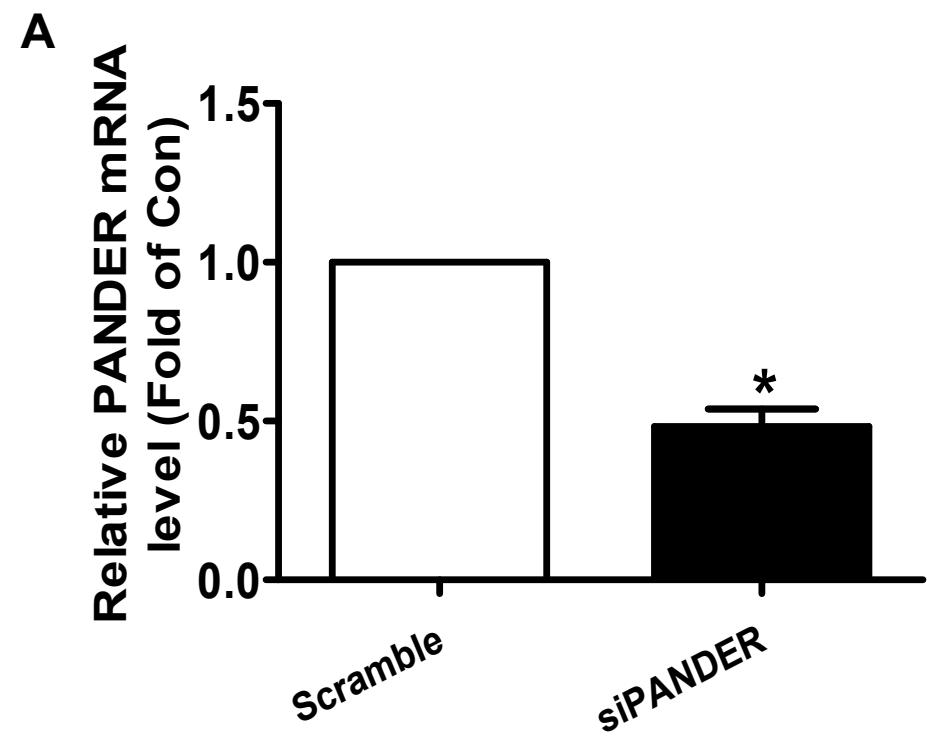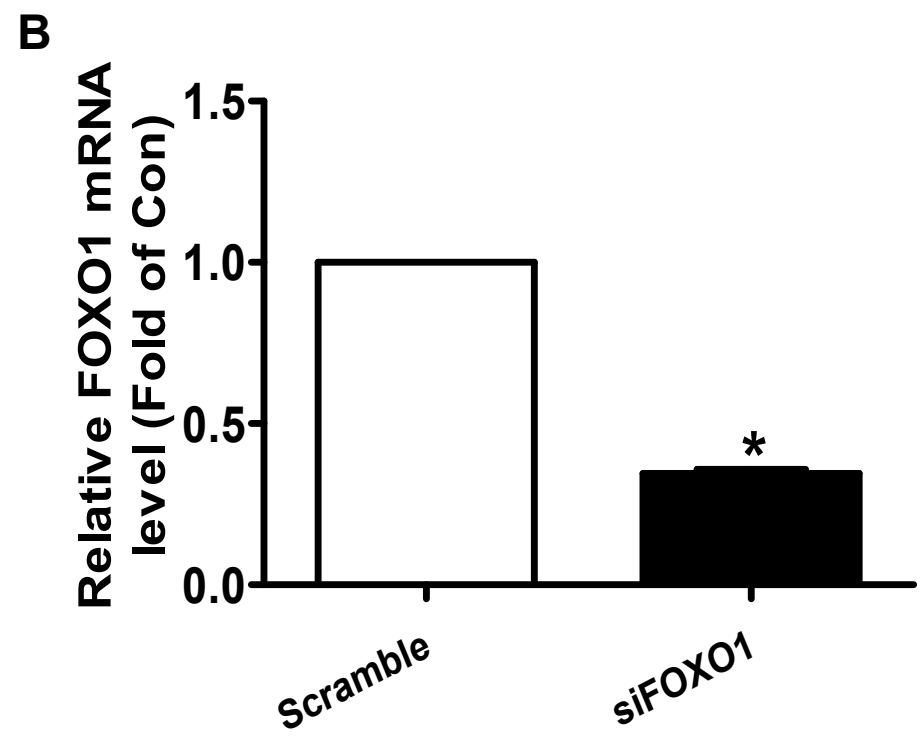

Supplement: Supplementary file 1 [file JCMM-23-1746-s001.pdf]
