# Supplementary material for: FAM3B (PANDER) functions as a co‐activator of FOXO1 to promote gluconeogenesis in hepatocytes
Source: J Cell Mol Med. 2018 Nov 28;23(3):1746–58. doi: 10.1111/jcmm.14073 (PMC6378191; doi:10.1111/jcmm.14073)
Supplement: Supplementary file 2 [file JCMM-23-1746-s002.docx]

**Supplemental table 1. List of** **oligonucleotide primer pairs used in real time RT-PCR analysis.**

| Target Gene | Sense Primer(5'-3') | Antisense Primer(5'-3') | Annealing temperature |
| --- | --- | --- | --- |
| β-actin(H) | AGCCATGTACGTAGCCATCC | GCTGTGGTGGTGAAGCTGTA | 60℃ |
| PEPCK(H) | CAAACTGCCCAAGATCTTCCA | CCATCGATCCGGTTGAACAT | 60℃ |
| G6Pase(H) | GGCATTGCTGTTGCAGAAACT | AGGTCTACACCCAGTCCCTTGA | 60℃ |
| FOXO1(H) | GGATGGTCAAGAGCGTGC | CTCGGCTTCGGCTCTTAG | 60℃ |
| PANDER(H) | CCATTGGCTGGTGGCCTG | GGGACTGGAGCTTTGAGGAC | 60℃ |

H：Human; If not indicated, all the primer sequences are referred to human.

**Supplemental table 2**. siRNA sequences against human PANDER and FOXO1 mRNAs

| Dulex Name | SenseSeq | AntiSeq |
| --- | --- | --- |
| PANDER-1 | GGACCGAUGACAAAGUUUATT | UAAACUUUGUCAUCGGUCCTT |
| PANDER-2 | GGUCUAGCUGGGUAUUUAUTT | AUAAAUACCCAGCUAGACCTT |
| PANDER-3 | GGGUAUUUAUUGCAGCAAATT | UUUGCUGCAAUAAAUACCCTT |
| PANDER-4 | CGAUGCCAAGAAUGCCAUATT | UAUGGCAUUCUUGGCAUCGTT |
| FOXO1-1 | GGAGGUAUGAGUCAGUAUATT | UAUACUGACUCAUACCUCCTT |
| FOXO1-2 | CGAGUUUAGUAACAGUGCATT | UGCACUGUUACUAAACUCGTT |
| FOXO1-3 | CCAAGUAGCCUGUUAUCAATT | UUGAUAACAGGCUACUUGGTT |
| FOXO1-4 | UCAUGUCUUGAUAAGUUAATT | UUAACUUAUCAAGACAUGATT |

**Suppl figure legends.**

**Figure 1. PANDER protein is present in both the cytoplasm and nucleus of mouse livers.** Confocal imaging revealed that PANDER protein is present in both cytoplasm and nucleus of mouse liver cells. Representative nucleuses had been indicated by arrows

**Figure 2. Efficacy of siRNAs against PANDER and FOXO1 in HepG2 cells.** Cells were transfected with 50nM siRNA mixtures or scrambled siRNA for 24 hours before analysis of target mRNA level. N=3, *P<0.05 versus control cells transfected with scrambled siRNA.
